# Red Queen dynamics in multi-host and multi-parasite interaction system

**DOI:** 10.1038/srep10004

**Published:** 2015-04-22

**Authors:** Jomar F. Rabajante, Jerrold M. Tubay, Takashi Uehara, Satoru Morita, Dieter Ebert, Jin Yoshimura

**Affiliations:** 1 Graduate School of Science and Technology, Shizuoka University, 3-5-1 Johoku, Naka-ku, Hamamatsu, 432-8561, Japan; 2 Mathematics Division, Institute of Mathematical Sciences and Physics, University of the Philippines Los Baños, College, Laguna 4031, Philippines; 3 Department of Mathematical and Systems Engineering, Shizuoka University, 3-5-1 Johoku, Naka-ku, Hamamatsu, 432-8561, Japan; 4 Zoological Institute, University of Basel, Vesalgasse 1, 4059 Basel, Switzerland; 5 Marine Biosystems Research Center, Chiba University, Uchiura, Kamogawa, Chiba 299-5502, Japan; 6 Department of Environmental and Forest Biology, State University of New York College of Environmental Science and Forestry, Syracuse, NY 13210 U.S.A

## Abstract

In host-parasite systems, dominant host types are expected to be eventually replaced by other hosts due to the elevated potency of their specific parasites. This leads to changes in the abundance of both hosts and parasites exhibiting cycles of alternating dominance called Red Queen dynamics. Host-parasite models with less than three hosts and parasites have been demonstrated to exhibit Red Queen cycles, but natural host-parasite interactions typically involve many host and parasite types resulting in an intractable system with many parameters. Here we present numerical simulations of Red Queen dynamics with more than ten hosts and specialist parasites under the condition of no super-host nor super-parasite. The parameter region where the Red Queen cycles arise contracts as the number of interacting host and parasite types increases. The interplay between inter-host competition and parasite infectivity influences the condition for the Red Queen dynamics. Relatively large host carrying capacity and intermediate rates of parasite mortality result in never-ending cycles of dominant types.

Population densities of antagonistic interacting species in host-parasite and predator-prey systems often exhibit oscillatory behavior, e.g., limit cycles and heteroclinic cycles^1^,^2^,^3^,^4^. In the case of host-parasite systems, oscillations with perpetual replacement of dominant hosts and parasites are expected because of differential susceptibility of hosts and differential infectivity of parasites^5^,^6^,^7^,^8^. This persistent replacement behavior is called Red Queen dynamics^9^,^10^. The non-equilibrium dynamics of alternating dominance are considered an important factor in maintaining biodiversity^11^,^12^.

Parasites infect preferably hosts with high abundance and a high degree of genetic uniformity, giving rare host types an advantage during host-parasite coevolution^13^,^14^,^15^,^16^. The perpetual replacement of dominant hosts in a multi-species or multiple-genotype system might disappear converging to a reduced interacting system (e.g., single host-single parasite) if there is an all-resistant host and a parasite being able to infect all hosts. In natural ecosystems, such super-hosts and super-parasites are scarcely observed, possibly because tradeoffs restrict their presence^15^,^16^,^17^,^18^. Indeed, natural host-parasite systems involve diverse hosts with respect to susceptibility and diverse parasites with respect to infectivity^19^,^20^. We therefore predict the occurrence of Red Queen dynamics in environments with various hosts and parasites as long as each species can avoid extinction and recover when parasites reach low density.

The Red Queen has been described to occur in distinct modes, namely, fluctuating Red Queen, escalatory Red Queen and chase Red Queen^21^,^22^,^23^. The Red Queen manifests in various experimental and *in silico* studies^9^,^14^,^21^,^22^,^23^, but there are no detailed studies and illustrations that demonstrate fluctuating Red Queen dynamics when the numbers of hosts and parasites exceed three. One of the manifestations of fluctuating Red Queen is the out-of-phase population cycles with perpetual replacement of dominant population that we describe in this paper.

Various mathematical models of competitive and antagonistic systems focus on three or less hosts and the equal number of parasites^19^,^20^,^24^,^25^. However, many natural host-parasite interactions usually involve multiple types of hosts and parasites^19^,^20^. In a first approximation, one may expect that the results of multiple-type models are qualitatively similar to those of two to three types. However, when we increase the number of types, the number of parameters in the models escalates (more than 100 in models with more than 10 host and 10 parasite types), the complexity of the interactions increase exponentially, and stochastic effects – including chance extinctions – become more severe. Therefore, it is unclear if multi-host and multi-parasite models give qualitatively similar prediction to more simple models. Thus, it is valuable to assess how the numbers of host and parasite types affect the occurrence and qualitative behavior of Red Queen dynamics. Identifying the conditions that give rise to Red Queen dynamics is useful for understanding host-parasite coevolution in natural systems.

We are interested to investigate Red Queen dynamics in interaction system with multi-host and multi-parasite types. The term ‘types’ usually means genotypes of a species but it can be extended to host and parasite varieties and even to different species by modifying the parameter conditions (e.g., growth rate and competitive ability) of each type differently. To evaluate the dynamics of many types, we investigate host-parasite population dynamics with up to 20 hosts and 20 parasites by means of computer simulations. As first attempt in modeling high-dimensional host-parasite systems, we focus on the consumer-resource model of host and parasite interactions with type-II functional response. We also limit our analyses to the parameter spaces with which Red Queen dynamics is more likely to be expected.

The Red Queen has two standard meanings. The first one explains host and parasite coexistence, extinction and biodiversity, following the theory of Van Valen^9^. The other meaning describes host-parasite dynamics based on negative frequency dependent selection, which can explain the advantage of sexual reproduction and genetic recombination^26^,^27^,^28^. In this paper, we define the Red Queen dynamics (or Red Queen cycles) as the case where oscillating host (parasite) densities exhibit dominance replacement by another hosts (parasites) such that the amplitude of the oscillations are qualitatively identical but out-of-phase. Every host (parasite) has the opportunity to be numerically dominant for a certain period of time. In line with the two standard meanings, our definition explains host-parasite dynamics with negative frequency dependent selection that results in no optimality but never-ending switching dominant types. In mathematical point-of-view, we can see Red Queen cycles as those converging towards heteroclinic cycle undergoing sequential heteroclinic switching. The sustained decline and recovery of populations that lead to the Red Queen cycles maintain the temporal diversity of hosts and parasites. This winnerless phenomenon is one of the many manifestations of the Red Queen^21^.

## Results

### Simulation preliminaries

We employ an ordinary differential equation (ODE) model and examine whether the long-term apparent dynamics result in Red Queen cycles. We numerically investigate the behavior of host-parasite interaction system with type-II functional response for *n* hosts and *n* parasites^29^,^30^. The population of a host type increases according to the effective growth rate and decreases with the parasitic infections, while that of a parasite decreases by a constant death and increases with parasitic utilization of hosts (numerical response). This model does not only include antagonistic interaction between hosts and parasites but also inter-host competition. A simple form of the model is given by the following ODE (for model assumptions, see supporting text in the Supplementary Information):
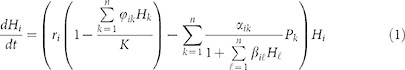

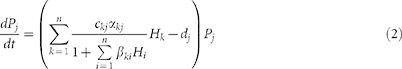

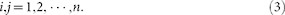
where *H_i_* and *P_j_* denote the population of host type (species, genotype, strain or variety) *i* and parasite type *j*, respectively. The parameters *r_i_*, *d_j_* and *K* denote the basal growth rate of host *i*, the death rate of parasite *j*, and the carrying capacity of hosts, respectively. The coefficients 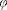
*_ik_*, *c_ij_* and *β_ik_* represent competitive ability between hosts, host-to-parasite conversion due to host utilization, and energy allotment of parasites to other host type, respectively. For simplicity, assume *r_i_ = r* for all *i*, *j* = *d_j_* for all *j*, 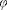
*_ik_ = 1* for all *i*,*k*, *c_ij_ = 1* for all *i*,*j*, and *β_ik_ = 0* for all *i≠k* (see Methods for the details of this assumption).

In the type-II functional response (*α_ij_H_i_/(1* *+* *H_i_)*), *α_ij_* is the parasitism efficiency of parasite *j* against host *i*. The fraction of the host density that one parasite can utilize tends to satiate as host population proliferates. In order to simulate the alternating dominance phenomenon, we set the parasitism efficiency matrix **A** = [*α_ij_*] to be diagonally dominant, such that
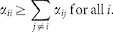
This diagonally dominant matrix shows that *H_i_* is the main host of parasite *P_j = i_*, while all *H_k≠i_* are minor alternatives. This means that there is no superior parasite (host) nor inferior parasite (host) in infectivity (parasite resistance). We hypothesize that replacement of dominant hosts in the Red Queen dynamics is expected to occur under this high specificity condition. Henceforth, we restrict our simulations under this condition.

For simplicity, this model assumes that the infected hosts are immediately eradicated as represented by the functional response in equation (1). This implies that an infected host has no ability to reproduce and does not recover from disease, as is often the case in bacteria infected with phages and in invertebrate hosts infected with castrating parasites.

### Simulation results

**Figure 1:**
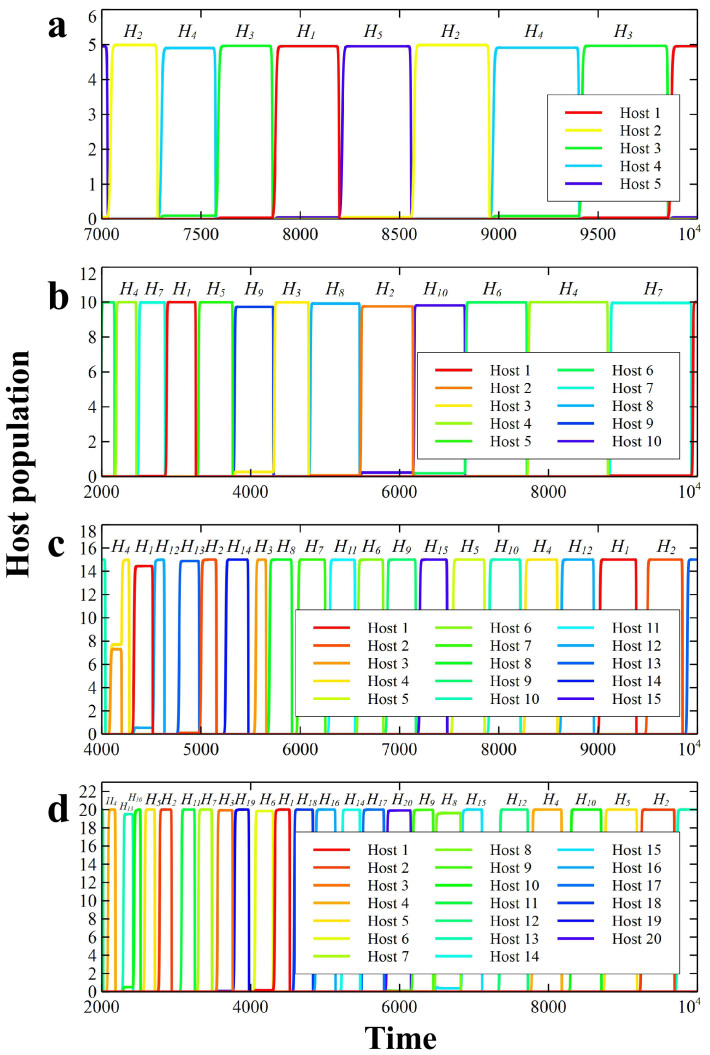
Illustrative examples of host population time series showing Red Queen dynamics (see the supporting text in the Supplementary Information for the parameter values, and Supplementary Fig. 1 for the associated parasite population time series). Note that the population densities of non-dominant type are not equal to zero. (a) With 5 hosts and 5 parasites. (b) With 10 hosts and 10 parasites. (c) With 15 hosts and 15 parasites. Notice that the order of dominance is not constant. For example, in one period *H_13_* replaces *H_12_* but in another period *H_1_* replaces *H_12_*. Moreover, the length of dominance of one population in a certain period can be longer than the previous period (e.g., see the graph of *H_12_*). (d) With 20 hosts and 20 parasites.

**Figure 2:**
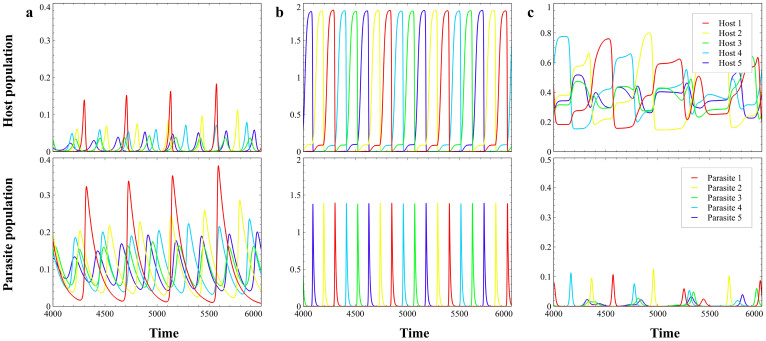
Illustrative examples of population time series showing Red Queen and non-Red Queen dynamics; *n = * 5, *K = * 2, *r = * 0.1, *α_ij_ = * 0.01 for *i ≠ j*, and *α_ij_ = * 0.96 for *i = j*. (a) Non-Red Queen dynamics in host populations where the minimum points of the oscillations are near relatively low values; *d = * 0.01. (b) Red Queen dynamics (out-of-phase population cycles with perpetual replacement of dominant population); *d = * 0.12. (c) Non-Red Queen dynamics exhibiting permanent coexistence in host populations (the population of all hosts are relatively far from zero); *d = * 0.27.

In our simulations, Red Queen dynamics emerge in antagonistic systems with type-II functional response involving more than three hosts and parasites, such as with 5, 10, 15 and 20 types (Fig. 1 and Supplementary Fig. 1). Only certain combinations of parameters lead to the Red Queen. For example, an intermediate or high level of host growth rate (*r*) supports Red Queen cycles. The Red Queen dynamics are also influenced by the mortality rate and infectivity of parasites, and carrying capacity of hosts. Intermediate degree of parasite death rate (*d*) favors Red Queen dynamics in host and parasite populations; whereas, a relative low or a relatively high death rate results in non-Red Queen cycles (Fig. 2). The oscillating population density of a certain host is influenced by the period of low density and recovery of its specialist parasite. The asynchronous low density and recovery in parasite populations stimulate replacement of dominant hosts (i.e., parasite drags down the dominant host).

**Figure 3:**
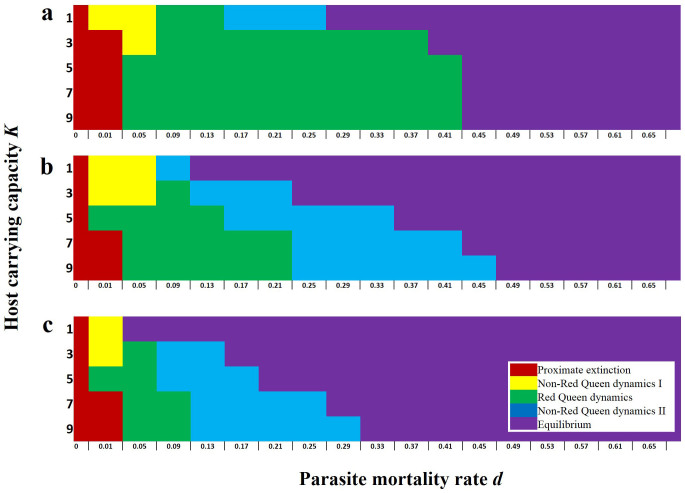
Qualitative behavior diagram of host populations when varying the parasite death rate *d* and host carrying capacity *K*; *r = * 0.5, *α_ij_ = * 0.01 for *i ≠ j*. Proximate extinction means hosts have long periods of minimal population sizes near edge of extinction due to high parasitism (Supplementary Fig. 2a). Non-Red Queen dynamics I are oscillations where the minimum points are near relatively low values while non-Red Queen dynamics II are those towards permanent coexistence in host populations (Fig. 2). Note that the qualitative classification of cycles at the boundary between Red Queen and non-Red Queen dynamics are often difficult to identify because transition events happen at these boundaries and due to lack of available computational methods for distinguishing the different cycles in a differential equation model with many variables and parameters. (a) With 3 hosts and 3 parasites. (b) With 10 hosts and 10 parasites. (c) With 20 hosts and 20 parasites.

In order to examine what conditions promote Red Queen dynamics, we varied the parasite death rate *d* and the host carrying capacity *K* (Fig. 3). We classify the different qualitative dynamics in Figure 3 by visual observation of the host population time series (note that we define Red Queen cycles as out-of-phase population cycles with perpetual replacement of dominant population as in Fig. 2b). The phase diagrams of 3 host types (Fig. 3a), 10 types (Fig. 3b) and 20 types (Fig. 3c) are qualitatively similar except that the parameter region that generates the Red Queen dynamics contracts as the number of hosts and parasites increases. The host extinction region (Supplementary Fig. 2a) appears in very low *d* and population equilibrium in regions of high *d* (Supplementary Fig. 2b). Non-Red Queen cycles I (Fig. 2a) appear near extinction region (low *d*) and low *K*, while the cycles II (Fig. 2c) next to equilibrium region (high *d*). As *K* increases, the phase transitions among Red Queen/non-Red Queen cycles and equilibrium shift to larger *d* values (Fig. 3). Thus, the Red Queen dynamics appear in a wider range of *d* when *K* is large. The value of *K* also affects the shape of the Red Queen cycles, such as the amplitude of cycles and the periodic length of dominance of hosts increase with *K*. The order and length of dominance are not necessarily constant in every period (e.g., see Fig. 1c).

Inter-host competition and a diagonally dominant parasitism matrix (**A** = [*α_ij_*]) are favorable for generating Red Queen cycles (Supplementary Figs. 3 and 4). In fact, if *α_ij_ = 0*, *i≠j* (i.e., no minor alternate hosts), the Red Queen dynamics still persist (Supplementary Fig. 4a). In contrast, if every *α_ij_* becomes nearly equal (i.e., *α_ij_≈α* for all *i*,*j*), the out-of-phase cycles diminish (Supplementary Fig. 4c). Finally, Red Queen dynamics still emerge even when some degrees of stochastic noise and differential parameter values are introduced to the model parameters (Supplementary Fig. 5 and 6). The Red Queen dynamics also emerge when the underlying inter-host competition model is modified (e.g., inter-host competition following a non-polynomial function. See Supplementary Fig. 7a). However, the presence and behavior of Red Queen cycles could change if some assumptions affecting parasitism are varied, such as employing a different functional response (Supplementary Fig. 7b). For example, in using type-III functional response (Supplementary Fig. 7b), it is possible that the Red Queen cycles occur only in at most two host/parasite types and the rest of the hosts and parasites remain rare. This is an example of an interaction system where the Red Queen dynamics are not able to include more than two host and parasite types.

## Discussion

Mathematical studies have shown the Red Queen dynamics in host-parasite systems with less than three interacting hosts and parasites but these systems may not be adequate to predict the dynamics involving many host and parasite types^19^,^20^,^24^,^25^. Our current extensive simulations show theoretical evidence that Red Queen dynamics still emerge in an antagonistic system with at least up to 20 hosts and 20 parasites as long as each host type can recover when it reaches very low densities (the edge of extinction). An increased growth potential of hosts provides each host the ability to recover from the adverse effect of parasites.

The Red Queen dynamics illustrate negative frequency dependent selection where a rare host genotype is favored by selection because the common or dominant genotype is infected by the prevailing parasite type. The rate of parasite mortality (*d*) is significant in this negative frequency dependent selection. If a certain host is dominant then its specialist parasite should have an intermediate degree of mortality rate to allow parasite proliferation. As the parasite population grows, the host population decreases because of infection. The decrease in the population density of this host allows other host types to increase and eventually dominate the system. This results in cyclic abundance of host and parasite types with parasites tracking their hosts.

An intermediate degree of parasite mortality provides a good condition to have asynchronous decline and recovery in host and parasite populations. The Red Queen cycles may disappear if the parasite death rate (*d*) is relatively low and relatively high (Fig. 2 and Supplementary Fig. 2). Minimal parasite mortality prompts slow recovery of the hosts resulting in proximate extinction of host populations (Supplementary Fig. 2a). Moderately low parasite mortality severely affects several host types leading to non-Red Queen cycles (Fig. 2a). Meanwhile, relatively high parasite death rate yields non-Red Queen dynamics (Fig. 2c) and equilibrium-converging host density (Supplementary Fig. 2b) because parasites may go extinct. The order of dominant types and length of population cycles are not necessarily constant in every period due to the inherent complexity of the interaction among many host and parasite types.

It should be noted that oscillations, not only Red Queen cycles, are important in generating biodiversity^12^. Non-Red Queen cycles can also be a mechanism driving variation. However, these non-Red Queen cycles are transition events between the Red Queen and equilibrium dynamics. Non-Red Queen dynamics may exhibit host/parasite cycles that do not have identical amplitude, which implies that some host and parasite types do not have similar opportunity for attaining high population sizes as the other types. In the Red Queen dynamics, the collective fitness of the hosts and parasites has constant pattern, as characterized by the identical population amplitude, even though the hosts and parasites continuously undergo negative frequency dependent selection. This is coherent with the Red Queen hypothesis, which states that hosts and parasites coevolve but their fitness stays the same.

Parasitism plays a big role in generating out-of-phase oscillatory behavior. Without parasites, the system reduces to a model of logistic inter-host competition that often converges to an equilibrium state. Nevertheless, parasitism alone is not enough to generate out-of-phase cycles required by the Red Queen dynamics but rather parasitism and inter-host competition need to occur together. Rivalry among hosts produces oscillations that stimulate the alternating replacement of dominant host population (Supplementary Fig. 3). In the Red Queen dynamics, the inter-host competition assimilates the cost of evolutionary selection against the common host type. The number of competing hosts dictates the necessary size of the carrying capacity that would induce the Red Queen cycles (Fig. 3). However, the carrying capacity should not be too large as unbounded value of *K* destabilizes the system, a phenomenon also observed in the Paradox of Enrichment^31^.

Evidence for parasite-mediated replacement of dominant host have been reported earlier^3^,^6^,^7^,^8^. There are also empirical supports for Red Queen dynamics showing the coevolution in host and parasite genotypes^26^,^27^,^28^,^32^,^33^. Hosts should be able to recover from the adverse effect of parasitism, else they would go extinct. Fossil records show examples of extinction, presumably because the organisms were not able to cope up with the coevolving antagonists^9^,^26^,^27^,^28^,^33^. In addition, Red Queen dynamics are widespread in models of host-parasite coevolution because the strength of selection is almost symmetrical. This is in contrast to predator-prey interaction with biased evolutionary arms races (‘life-dinner’ principle), which results in a limited potential for Red Queen dynamics^21^.

Furthermore, a basic requirement for Red Queen dynamics is a high degree of specificity in the interaction of hosts and parasites^34^,^35^,^36^. Low degree of specificity results in a loss of diversity. Loss of host diversity can cause increased risk of disease (the monoculture effect), which is not followed by replacement of dominant type^14^,^15^,^16^. A diagonally dominant parasitism matrix (**A** = [*α_ij_*]), which implies the absence of superior host and parasite, is essential in generating out-of-phase cycles (Supplementary Fig. 4). Limitless advantage of one host/parasite type against another type is not real in nature; rather biological trade-offs seem to prevail^37^. Trade-offs constrain the adaptive ability of species and thus can lead to genetic diversity^18^. In an antagonistic system with many host and parasite types, the Red Queen phenomenon is a consequence of the presence of diverse host types and their specialist parasites.

The parasitism efficiency matrix can be seen as a representation of the continuum between gene-for-gene and matching-alleles systems^38^. A parasitism efficiency matrix that accepts universal virulence (e.g., non-zero matrix with equal elements) resembles a pure gene-for-gene model. Whereas, a host-parasite interaction with diagonally dominant parasitism efficiency matrix characterizes a matching-alleles model. It denotes a pure matching-alleles model if the elements of the upper and lower triangular of the matrix are zeroes and the elements of the diagonal are non-zeroes. Red Queen theories involving matching-alleles models are widespread in host-parasite interaction^38^. Furthermore, the matrix representing the case between pure gene-for-gene and pure matching-alleles systems is called a partial matching-alleles model (or partial gene-for-gene model, depending on the focus). Equation (1) and (2) with a diagonally dominant matrix having non-zero upper and lower triangular is an example of such partial model. The partial model based on an asymmetrical multilocus gene-for-gene system also exhibits endless Red Queen coevolutionary cycle^22^.

One of the most important observation in our study is the contraction of the parameter region where Red Queen dynamics occur as the number of interacting host and parasite types increases (Fig. 3). This contraction is primarily due to the effect of parasite mortality rate (*d*). If the number of interacting host types increases then the competition among hosts intensifies. This inter-host competition affects the population growth of each host, which in turn influences the parasites. Since parasites depend on hosts, then each parasite needs to offset the population decline of their major host to maintain out-of-phase population cycles. Hence, a higher parasite mortality rate does not sustain the Red Queen dynamics, resulting in the contraction of the Red Queen dynamics parameter region (Fig. 3).

In general, the qualitative behavior of the solution to equations (1) and (2) describes various antagonistic interaction, such as host-parasite, predator-prey and exploiter-victim^39^. The emphasis of our discussion is on host-parasite interaction because the Red Queen has been exceptionally common to this type of interaction^21^. We focus on showing how the ecological interaction of parasites as consumers and hosts as resources results in the Red Queen dynamics^40^. The interaction of multi-type bacteria and their multi-type virus (phages) can be modeled by such consumer-resource model^41^,^42^,^43^. The ‘killing the winner’ dynamics in bacteriophage predation is associated with the winnerless Red Queen cycles^41^,^42^,^43^,^44^. Moreover, equations (1) and (2) are simplified version of the standard disease model, such as the Susceptible-Infected model^40^. The gross growth rate of hosts in equation (1) does not account for infected hosts because the infected hosts are assumed to be immediately eliminated from the population. The results of our simulation are limited to host-parasite interaction where there is no recovery and no reproduction of the infected hosts. We also limit our investigation to type-II functional response. In future studies, other epidemiological models and functional response curves (types I and III) can be employed to further investigate the different modes of the Red Queen occurring in multi-host and multi-parasite interaction system^21^,^40^,^45^.

In conclusion, the number of interacting hosts and parasites as well as the dynamic interface between parasitism and inter-host competition in a host-parasite system are essential in generating Red Queen dynamics (Fig. 3). For certain conditions, the perpetual replacement of dominant host and parasite types is guaranteed by the oscillating presence (in the form of alternating low density and recovery) of diverse hosts and specialist parasites. One of the important characteristics of the Red Queen dynamics is that it preserves the temporal diversity of species. This temporal biodiversity does not necessarily involve the coexistence of species types in a certain period of time but rather biodiversity of species through time^26^,^27^. Understanding the dynamics of host-parasite interaction can provide new insight into the fields of evolutionary biology, parasite ecology, biomedical parasitology and epidemiology^16^,^19^,^26^,^32^. Our findings have significant impact on the theory of host-parasite co-evolution, especially in extending current studies to a multiple-genotype or many-species system.

## Methods

A mathematical model with many variables and arbitrary parameters is difficult to analyze. Hence, we search for the Red Queen dynamics using intensive simulation but in a restricted condition (*r_i_ = r* for all *i*, *j = d_j_* for all *j*, 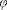
*_ik_ = 1* for all *i*,*k*, *c_ij_ = 1* for all *i*,*j*, and *β_ik_ = 0* for all *i ≠ k*). We use a symmetric and deterministic model to investigate the basic dynamics of the Red Queen but we perform several strategies to break the symmetry and to relax the determinism of the model. We avoid the effect of symmetry by setting the initial condition to *H_i_(0) = H + 0.001(i* − *1)* for all *i* and *P_j_(0) = P + 0.001(j* − *1)* for all *j*. An unequal initial condition coupled with a diagonally dominant parasitism efficiency matrix (differential host susceptibility) results in nonidentical host mortality due to parasitism. This implies that the net growth of each host is not necessarily identical to the other host types even though the gross growth rate *r_i_ = r* for all *i*. The symmetric system can be used to easily find Red Queen cycles in systems with differential parameter values (Supplementary Fig. 6). Moreover, we use *H = P = 0.01* as default value in all the figures in this manuscript, as the initial values *H* and *P* do not affect our classification of Red Queen and non-Red Queen cycles.

The simplifying deterministic assumptions are relaxed by employing parameter perturbation using stochastic noise. We do sensitivity analysis by incorporating the following noisy parameters: *0* ≤ *randN(r_i_,σr_i_)* ≤ *1* as replacement for the deterministic *r_i_*, *0* ≤ *randN(d_j_,σd_j_)* ≤ *1* for *d_j_, 0* ≤ *randN(α_ij_,σα_ij_)* for *α_ij_*, *0* ≤ *randN(c_ij_,σc_ij_)* for *c_ij_*, *0* ≤ *randN(*
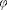
*_ik_*,σ
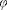
*_ik_)* for 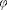
*_ik_*, and *0* ≤ *randN(α_ik_,σα_ik_)* for *β_ik_*, where *randN(μ,σμ)* is a normal random number with mean *μ* and standard deviation *σμ*.

The Red Queen dynamics can be considered as a special type of heteroclinic oscillations^46^, which are out-of-phase and have identical amplitude. There are existing mathematical and computational techniques for investigating heteroclinic cycles and bifurcation^46^,^47^. However, the complexities of finding and analyzing the qualitative behavior of equilibrium points, limit cycles and heteroclinic cycles increase with the number of variables and parameters. Since our model considers more than three variables and parameters involving two kinds of species (the hosts and the parasites) multiplied by the number of types, these measures become extremely complicated, cumbersome and unreliable. Hence, we simply classify the different qualitative dynamics and heteroclinic Red Queen oscillations by visual observation of the population time series (Fig. 3). In any case, by using computational method or by visual observation, the general qualitative behavior arising from the host-parasite model are maintained.

We use type-II functional response in the model of host-parasite interaction. The type-II functional response has been used by several empirical and theoretical studies of host-parasites interaction^23^,^48^,^49^,^50^. In Supplementary Figure 7, we modify the underlying inter-host competition model^51^ and the functional response to inspect the possible changes in the behavior of the Red Queen dynamics. In addition, the system of ODEs, equation (1) and (2), is solved using Runge-Kutta 4 with 0.01 as stepsize. In our search for the conditions that generate the Red Queen dynamics, we consider the following range of parameter values: *n* is from 2 to 20, *r_i_* is from 0.01 to 1, *d_j_* is from 0.001 to 1 and *K* is from 0.5 to 100. The deterministic parasitism efficiency matrix **A** *= * [*a_ij_*] is assumed to be diagonally dominant and symmetric where the sum of each row (column) is equal to 1, such that *α_ii_ = α + 1-αn* and *α_ij_ = α* for *i≠j*. The details of the model assumptions and parameter perturbation are discussed in the supporting text in the Supplementary Information.

## Author Contributions

J.F.R., D.E. and J.Y. conceived the study. J.F.R. and J.Y. built the model. J.M.T., T.U. and S.M. contributed to the programming of the model. J.F.R. ran the simulations. J.F.R., J.M.T., D.E. and J.Y. wrote the manuscript. J.F.R. is the lead author. All authors reviewed the manuscript.

## Supplementary Material

Supplementary Information Supplementary Information
