# Supplementary material for: Red Queen dynamics in multi-host and multi-parasite interaction system
Source: Sci Rep. 2015 Apr 22;5:10004. doi: 10.1038/srep10004 (PMC4405699; doi:10.1038/srep10004)
Supplement: Supplementary Information [file srep10004-s1.pdf]

Supplementary Information for

Red Queen dynamics in multi-host and multi-parasite interaction system

Jomar F. Rabajante, Jerrold M. Tubay, Takashi Uehara, Satoru Morita, Dieter Ebert, Jin Yoshimura

correspondence to: Jin Yoshimura (jin@sys.eng.shizuoka.ac.jp)

This PDF file includes:

Supporting text
Additional captions for Figures 1 to 3
Supplementary Figures 1 to 7

Supporting text

Details of the mathematical model

The ordinary differential equation (ODE) representation of the multi-type host-parasite system is

$$\frac{dH_i}{dt} = U_i H_i, i = 1,2,\cdots,m \quad \text{(Eq. S1)}$$

$$\frac{dP_j}{dt} = V_j P_j, j = 1,2,\cdots,n \quad \text{(Eq. S2)}$$

where the fitness functions are defined as

$$U_i = G_i - \sum_{k=1}^{n} f_{ik} P_k \quad \text{(Eq. S3)}$$

$$V_j = \sum_{k=1}^{m} c_{kj} f_{kj} H_k - d_j . \quad \text{(Eq. S4)}$$

The population of a host type increases according to the effective growth rate (G_i) and decreases with the parasitic infections (functional response). The population of a parasite decreases by a constant death (d_j) and increases with parasitic utilization of hosts (numerical response).

Definition of state variables and parameters in the general model

m	number of different host types (species/genotypes/varieties)
n	number of different parasite types (species/genotypes/varieties)
H_i	population size of host i
P_j	population size of parasite j

U_i	fitness function of host i
V_j	fitness function of parasite j
G_i	effective growth rate of host i
d_j	constant death rate of parasite j
f_{ij}	the parasitic utilization efficiency (part of the functional response)
c_{ij}	host-to-parasite conversion coefficient due to host utilization

Definition of parameters in the growth rate and functional response

r_i	basal growth rate of host i
φ_{ik}	relative strength of host i over host k in the inter-host competition
K	size of the carrying capacity of the environment of host populations affecting inter-host competition
α_{ij}	maximal infection rate or the efficiency of parasite j in infecting host i. The matrix containing the α_{ij}'s is called the parasitism efficiency matrix $\mathbf{A}=[\alpha_{ij}]$.
β_{ik}	coefficient representing the energy allotment of parasites to other host types

Model assumptions

We consider the following simplifying assumptions in the deterministic ODE system to investigate the basic dynamics of the Red Queen:

- We assume equal number of host and parasite types, $m=n$, so that each host can have a unique specialist parasite.
- We assume equal host gross growth rates ($r_i=r$ for all i) and equal parasite death rates ($d_j=d$ for all j). The host types have the same characteristics (such as growth rate) but differ in susceptibility to parasites. Each parasite also shares the same characteristics (such as death rate) but targets different principal host. Note that the Red Queen dynamics still arise when these assumptions are relaxed.
- We assume logistic growth for host populations with carrying capacity K. Let

$$G_i = r_i\left(1-\frac{\sum_{k=1}^{m}\varphi_{ik}H_k}{K}\right) \text{ where } \varphi_{ik}=1. \qquad \text{(Eq. S5)}$$

  Host i competes with host k if $\varphi_{ik}>0$. On the other hand, there is no inter-host competition between host i and host k if $\varphi_{ik}=0$ ($i\neq k$).

- We suppose $c_{ij}=1$ for all i and j. That is, one parasite can produce $\sum_{k=1}^{m} f_{kj} H_k$ number of new parasites by infecting an equal number $\left(\sum_{k=1}^{m} f_{kj} H_k\right)$ of hosts.
- We consider a generalized Holling-type II (hyperbolic) functional response where

$$f_{ik} = \frac{\alpha_{ik}}{1+\sum_{k=1}^{m} \beta_{ik} H_k} . \qquad \text{(Eq. S6)}$$

  We assume $\beta_{ii}=1$ and $\beta_{ik}=0$ for all $k \neq i$, which implies that the densities of other hosts are marginal to alter the functional response curve for infecting host i. Red Queen is still possible when this assumption is relaxed (see Supplementary Fig. 5 where the value of β_{ik} is random with average α_{ik}).
- The parasitism efficiency matrix **A**=[a_{ij}] is diagonally dominant and symmetric, where

$$\sum_{k=1}^{n} \alpha_{ik} = \sum_{k=1}^{n} \alpha_{kj} = 1 \text{ for all } i,j \text{ (note: } m=n\text{).} \qquad \text{(Eq. S7)}$$

  Specifically, we set $\alpha_{ij}=\alpha$ for $i \neq j$ and $\alpha_{ij}=\alpha+1-\alpha n$ for $i=j$. We hypothesize that differential susceptibility of hosts (infectivity of parasites) results in dominance replacement under this condition.
- The initial values are set to $H_i(0)=H+0.001(i-1)$ for all i and $P_j(0)=P+0.001(j-1)$ for all j to avoid unstable symmetric behavior. Without losing essential qualitative dynamics, we use $H=P=0.01$ in all the figures in the manuscript.

We define the Red Queen dynamics (or Red Queen cycles) as the case where all oscillating host/parasite densities exhibit dominance replacement by another host/parasite such that the amplitude of the oscillations are qualitatively identical but out-of-phase. Every host/parasite has the opportunity to be the sole dominant for a certain period of time. On the other hand, we generally refer to other types of oscillatory behavior as non-Red Queen dynamics.

Notes about the functional response: Type-I functional response denotes linear parasitic utilization of hosts. Type-II denotes parasitic utilization of hosts following a hyperbolic function. Type-III denotes parasitic utilization of hosts following a sigmoidal (S-curve) function. The curves of type-II and type-III converge to a saturation level; however, type-III includes parasite learning.

Numerical method

The system of ODEs is solved using Runge-Kutta 4 with stepsize=0.01. We use Berkeley Madonna (www.berkeleymadonna.com) to carry-out the computations. Maximum simulation time is $t=30,000$.

Details of the intensive simulation

In our search for the conditions that generate the Red Queen dynamics under the simplifying assumptions discussed above, we consider the following range of parameter values:

n : *2 to 20*

r_i : *0.01 to 1* (simulation increment=0.01)

d_j : *0.001 to 1* (simulation increment=0.001)

K : *0.5 to 100* (simulation increment=0.5).

In Supplementary Figs. 4 to 6, we use different sets of parameter values to illustrate the behavior of the system when the simplifying symmetric and deterministic assumptions are relaxed.

Parameter perturbation

Our deterministic simulations use symmetric parameter values (e.g., $r_i=r$ for all i and $d_j=d$ for all j). Here we show that the Red Queen dynamics is still possible when stochastic noise is present in the parameters. We suppose the following random values:

r_i : $0 \leq randN(r_i, \sigma r_i) \leq 1$

d_j : $0 \leq randN(d_j, \sigma d_j) \leq 1$

α_{ij} : $0 \leq randN(\alpha_{ij}, \sigma\alpha_{ij})$

c_{ij} : $0 \leq randN(c_{ij}, \sigma c_{ij})$

φ_{ik} : $0 \leq randN(\varphi_{ik}, \sigma\varphi_{ik})$

β_{ik} : $0 \leq randN(\alpha_{ik}, \sigma\alpha_{ik})$

where $randN(\mu, \sigma\mu)$ is a normal random number with mean μ and standard deviation $\sigma\mu$.

Additional captions for Figures 1 to 3

Parameter values for figures in the main text

Fig. 1. Illustrative examples of host population time series showing Red Queen dynamics.

(**a**) $n=5$, $K=5$, $r=0.3$, $d=0.2$, $\alpha_{ij}=0.01$ for $i\neq j$ and $\alpha_{ij}=0.96$ for $i=j$, $H_i(0)=0.01+0.001(i-1)$ for $i=1,2,\ldots,5$ and $P_j(0)=0.01+0.001(j-1)$ for $j=1,2,\ldots,5$.

(**b**) $n=10$, $K=10$, $r=0.3$, $d=0.15$, $\alpha_{ij}=0.01$ for $i\neq j$ and $\alpha_{ij}=0.91$ for $i=j$, $H_i(0)=0.01+0.001(i-1)$ for $i=1,2,\ldots,10$ and $P_j(0)=0.01+0.001(j-1)$ for $j=1,2,\ldots,10$.

(**c**) $n=15$, $K=15$, $r=0.3$, $d=0.07$, $\alpha_{ij}=0.01$ for $i\neq j$ and $\alpha_{ij}=0.86$ for $i=j$, $H_i(0)=0.01+0.001(i-1)$ for $i=1,2,\ldots,15$ and $P_j(0)=0.01+0.001(j-1)$ for $j=1,2,\ldots,15$.

(**d**) $n=20$, $K=20$, $r=0.3$, $d=0.06$, $\alpha_{ij}=0.01$ for $i\neq j$ and $\alpha_{ij}=0.81$ for $i=j$, $H_i(0)=0.01+0.001(i-1)$ for $i=1,2,\ldots,20$ and $P_j(0)=0.01+0.001(j-1)$ for $j=1,2,\ldots,20$.

Fig. 2. Illustrative examples of population time series showing Red Queen and non-Red Queen dynamics; $n=5$, $K=2$, $r=0.1$, $\alpha_{ij}=0.01$ for $i\neq j$, $\alpha_{ij}=0.96$ for $i=j$, $H_i(0)=0.01+0.001(i-1)$ for $i=1,2,\ldots,5$ and $P_j(0)=0.01+0.001(j-1)$ for $j=1,2,\ldots,5$.

(**a**) Non-Red Queen dynamics in host populations where the minimum points of the oscillations (trough) are near relatively low values; $d=0.01$.

(**b**) Red Queen dynamics; $d=0.12$.

(**c**) Non-Red Queen dynamics in host populations exhibiting permanent coexistence (the minimum points of the oscillations have relatively high values); $d=0.27$.

Fig. 3. Estimated qualitative behavior of host population when varying the parasite death rate d and host carrying capacity K. Note that the contraction of the parameter region of the Red Queen dynamics as the number of types (n) increases is not primarily due to the decreasing value of α_{ii} (the decreasing value of α_{ii} is from our assumption $\sum_{k=1}^{n}\alpha_{ik} = \sum_{k=1}^{n}\alpha_{kj} = 1$ for all i,j). We relaxed the assumption by setting $\alpha_{ii}=1$ even if $\alpha_{ij}=0.01$ for all $i\neq j$, and the results still show contraction of the parameter region.

(**a**) $n=3$, $r=0.5$, $\alpha_{ij}=0.01$ for $i\neq j$ and $\alpha_{ij}=0.98$ for $i=j$, $H_i(0)=0.01+0.001(i-1)$ for $i=1,2,3$ and $P_j(0)=0.01+0.001(j-1)$ for $j=1,2,3$.

Parasitism efficiency matrix for Fig. 3a (3 hosts, 3 parasites):

$$\begin{bmatrix} 0.98 & 0.01 & 0.01 \\ 0.01 & 0.98 & 0.01 \\ 0.01 & 0.01 & 0.98 \end{bmatrix}$$

(**b**) $n=10$, $r=0.5$, $\alpha_{ij}=0.01$ for $i\neq j$ and $\alpha_{ij}=0.91$ for $i=j$, $H_i(0)=0.01+0.001(i-1)$ for $i=1,2,\ldots,10$ and $P_j(0)=0.01+0.001(j-1)$ for $j=1,2,\ldots,10$.

Parasitism efficiency matrix for Fig. 3b (10 hosts, 10 parasites):

$$\begin{bmatrix} 0.91 & 0.01 & \cdots & 0.01 \\ 0.01 & 0.91 & \cdots & 0.01 \\ \vdots & \vdots & \ddots & \vdots \\ 0.01 & 0.01 & \cdots & 0.91 \end{bmatrix}$$

(**c**) $n=20$, $r=0.5$, $\alpha_{ij}=0.01$ for $i\neq j$ and $\alpha_{ij}=0.81$ for $i=j$, $H_i(0)=0.01+0.001(i-1)$ for $i=1,2,\ldots,20$ and $P_j(0)=0.01+0.001(j-1)$ for $j=1,2,\ldots,20$.

Parasitism efficiency matrix for Fig. 3c (20 hosts, 20 parasites):

$$\begin{bmatrix} 0.81 & 0.01 & \cdots & 0.01 \\ 0.01 & 0.81 & \cdots & 0.01 \\ \vdots & \vdots & \ddots & \vdots \\ 0.01 & 0.01 & \cdots & 0.81 \end{bmatrix}$$

Supplementary Figures 1 to 7

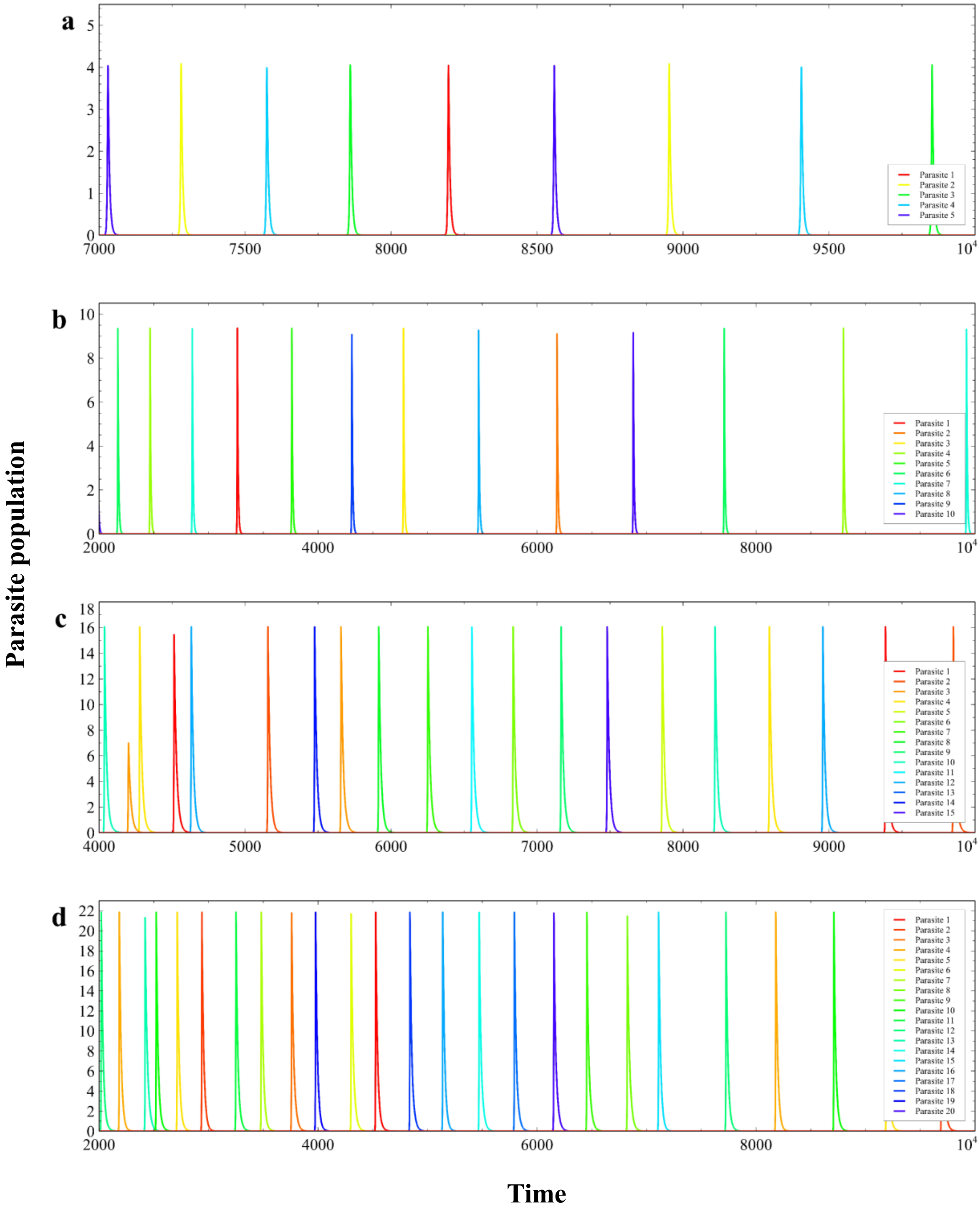

Supplementary Fig. 1. Illustrative examples of parasite population time series showing Red Queen dynamics. These are the parasites associated with the hosts in Fig. 1. **(a)** *n=5*. **(b)** *n=10*. **(c)** *n=15*. **(d)** *n=20*.

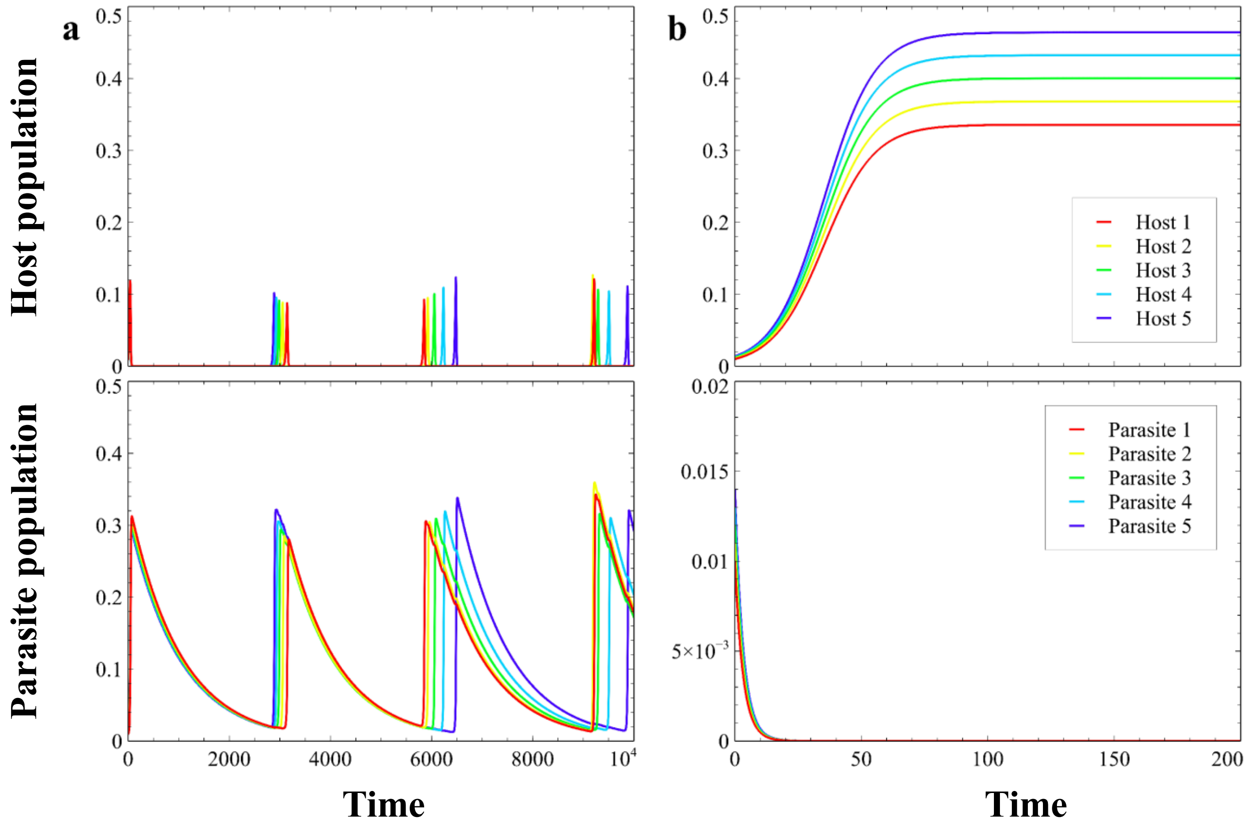

Supplementary Fig. 2. Host and parasite population time series showing proximate extinction and equilibrium scenarios, $n=5$, $K=2$, $r=0.1$ and $\alpha=0.01$. **(a)** Hosts have long periods of very low population sizes (proximate extinction in host populations), $d=0.001$. Mathematically, host extinction is an unstable equilibrium; however, a population density near zero increases the risk of extinction. **(b)** Equilibrium-converging time series, $d=0.32$.

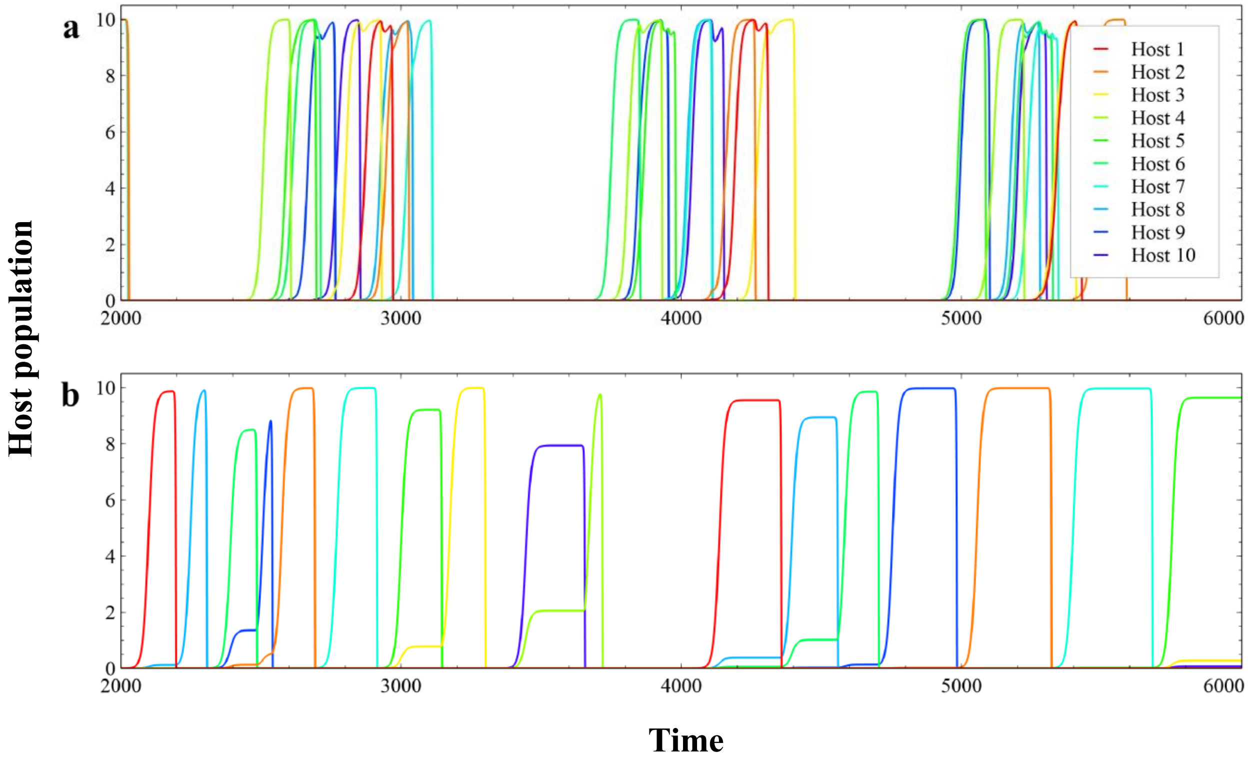

Supplementary Fig. 3. Host population time series illustrating the effect of inter-host competition in the formation of out-of-phase cycles, *n=10*, *K=10*, *r=0.1*, *d=0.1* and *α=0.01*. (**a**) Without inter-host competition. (**b**) With inter-host competition, out-of-phase cycles are formed.

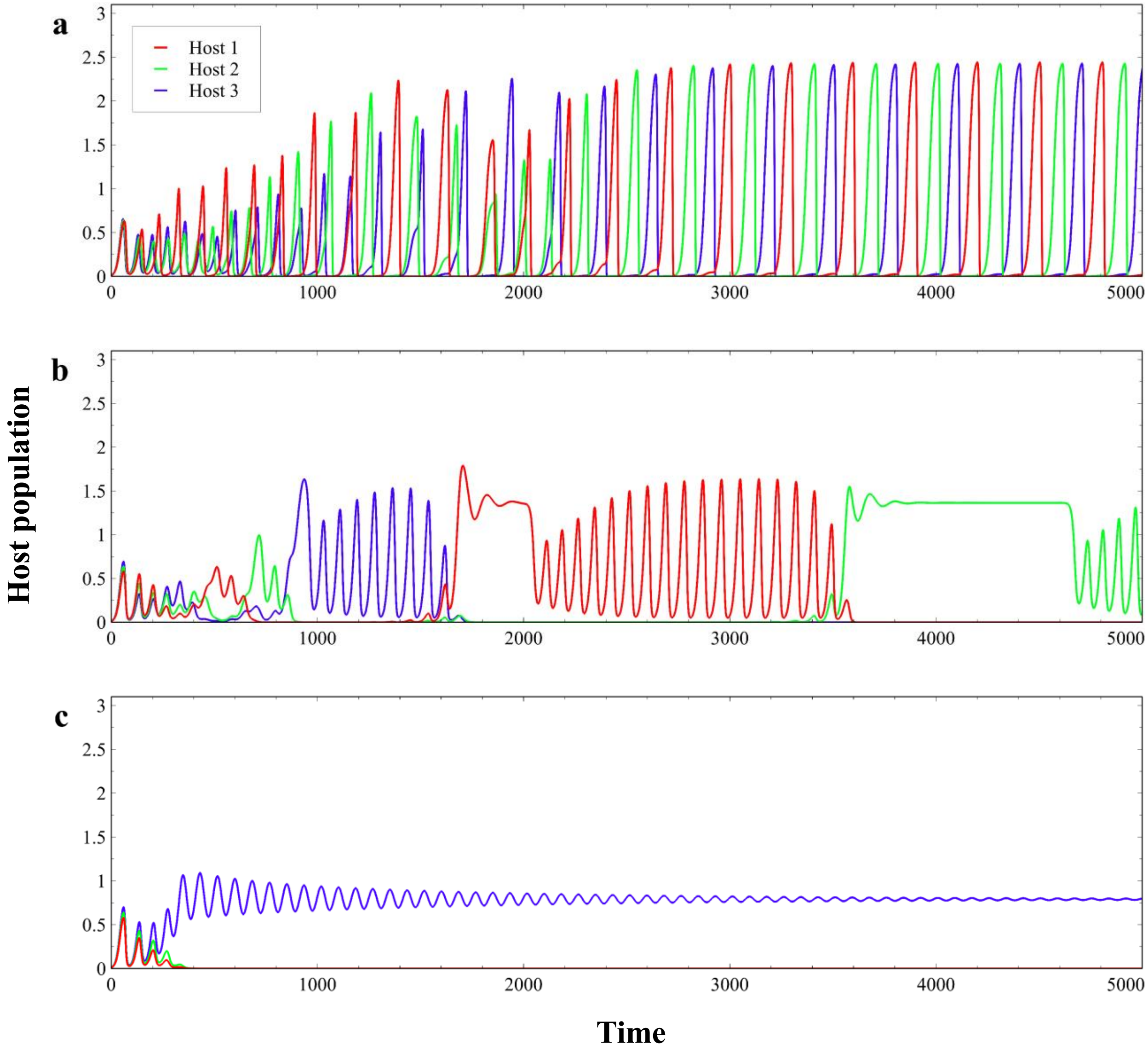

Supplementary Fig. 4. Low degree of specificity diminishes the Red Queen dynamics. An illustration of the effect of parasitism efficiency by varying the value of α; $n=3$, $K=2.5$, $r=0.1$ and $d=0.15$. (**a**) Absolute specificity, $\alpha=0$. A diagonally dominant parasitism matrix is favorable for the Red Queen dynamics to occur. (**b**) Intermediate specificity, $\alpha=0.26$. (**c**) Low degree of specificity, $\alpha=0.33$. When every α_{ij} becomes nearly equal, the out-of-phase cycles diminish and host populations with low fitness die out.

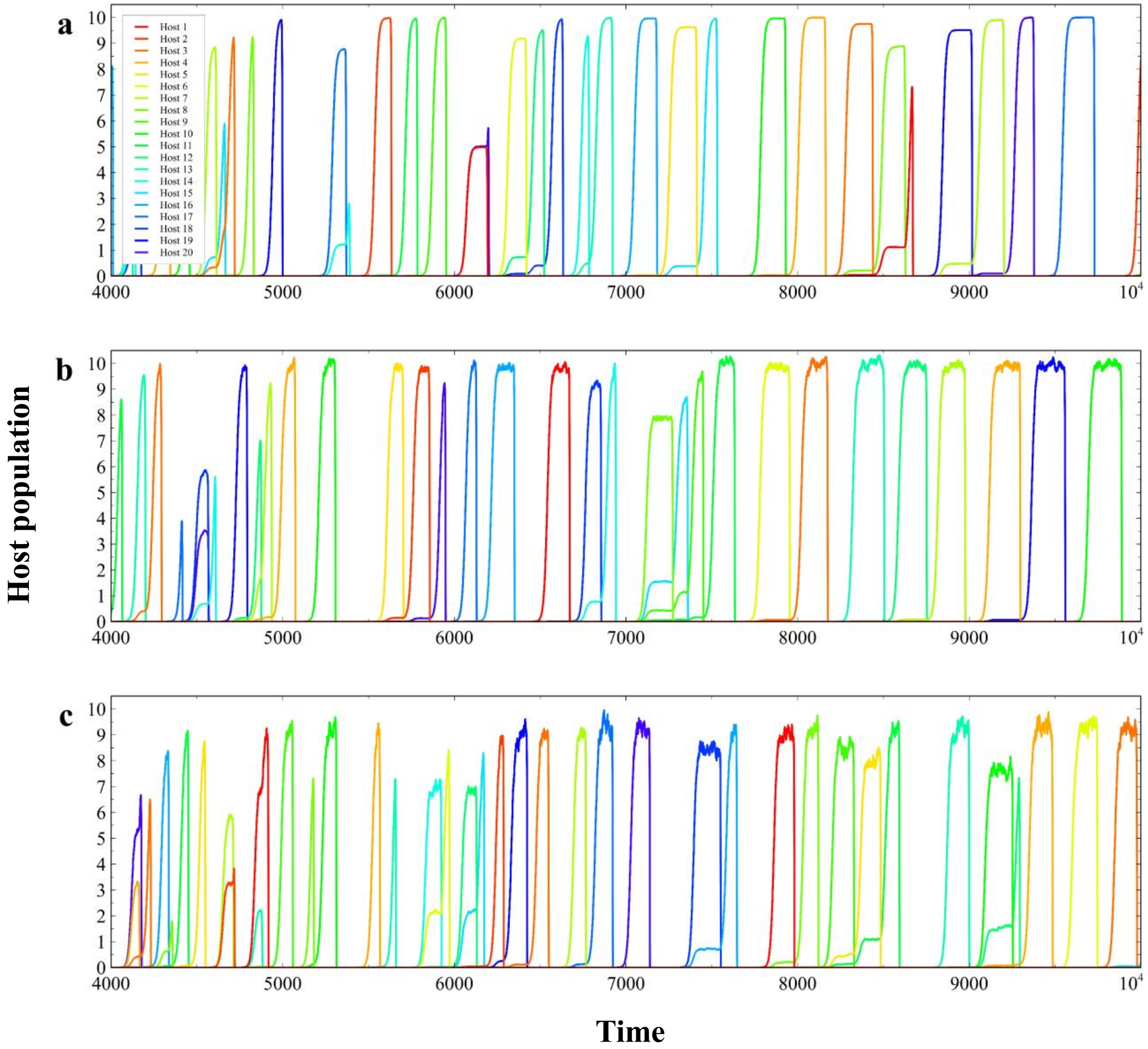

Supplementary Fig. 5. Sample paths showing dominance replacement in 20 host populations with noisy parameters, $K=10$. The mean values are $r_i=0.1$ for all i, $d_j=0.05$ for all j, $\alpha_{ij}=0.01$ for $i\neq j$, $\alpha_{ij}=0.81$ for $i=j$, $c_{ij}=1$ for all i,j, $\varphi_{ik}=\varphi_{ki}=1$ for all $i, k=1,2,\ldots,20$, and $\beta_{ik}=\alpha_{ik}$ for all $i, k=1,2,\ldots,20$. (**a**) Coefficient of variation $\sigma=0$ (deterministic). (**b**) Coefficient of variation $\sigma=0.5$. (**c**) Coefficient of variation $\sigma=1$.

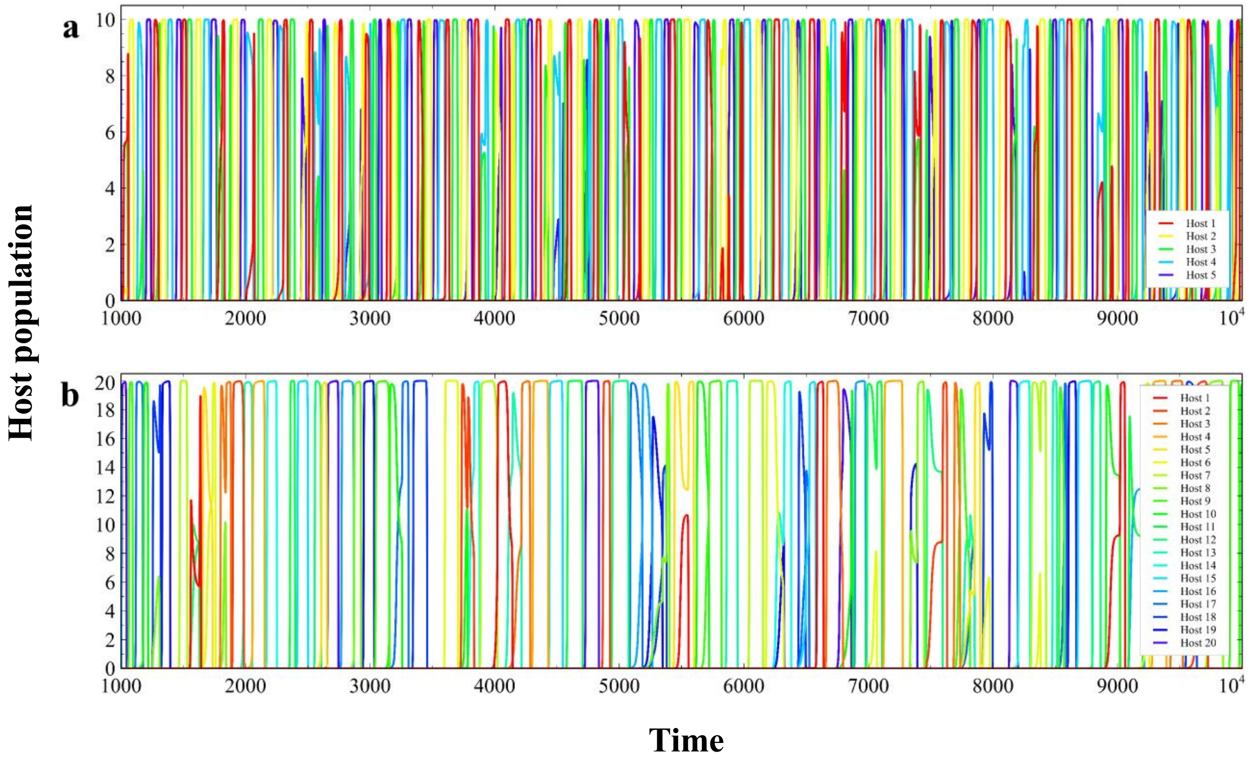

Supplementary Fig. 6. Red Queen dynamics occurring in a deterministic host-parasite system with differential values of r_i, d_j and φ_{ik}. The host types as well as the parasite types may represent different species. The matrix $[\varphi_{ik}]$ in the simulations has strictly positive eigenvalues. The host population time series are shown in the figure. (**a**) $n=5$. (**b**) $n=20$.

Notes:

Parameter values used in Supplementary Fig. 6a: $n=5$, $r_1=0.89$, $r_2=0.78$, $r_3=0.95$, $r_4=0.72$, $r_5=0.85$, $d_1=0.152$, $d_2=0.151$, $d_3=0.157$, $d_4=0.155$, $d_5=0.15$, $K=10$, $\alpha_{ij}=0.01$ for $i \neq j$ and $\alpha_{ij}=0.96$ for $i=j$, $H_i(0)=0.01+0.001(i-1)$ for $i=1,2,\ldots,5$ and $P_j(0)=0.01+0.001(j-1)$ for $j=1,2,\ldots,5$. The matrix $[\varphi_{ik}]$ is as follows

$$\begin{bmatrix} 1 & 0.89 & 0.72 & 0.95 & 0.87 \\ 0.89 & 1 & 0.91 & 0.92 & 0.77 \\ 0.72 & 0.91 & 1 & 0.85 & 0.71 \\ 0.95 & 0.92 & 0.85 & 1 & 0.94 \\ 0.87 & 0.77 & 0.71 & 0.94 & 1 \end{bmatrix}$$

Parameter values used in Supplementary Fig. 6b: $n=20$, $r_i=0.7+0.01(i-1)$, $d_j=0.05+0.0001(20-j)$, $K=20$, $\alpha_{ij}=0.01$ for $i \neq j$ and $\alpha_{ij}=0.81$ for $i=j$, $H_i(0)=0.01+0.001(i-1)$ for $i=1,2,\ldots,20$ and $P_j(0)=0.01+0.001(j-1)$ for $j=1,2,\ldots,20$. The matrix $[\varphi_{ik}]$ is as follows

$$
\begin{bmatrix}
1 & 0.72 & 0.73 & 0.74 & 0.75 & 0.76 & 0.77 & 0.78 & 0.79 & 0.8 & 0.81 & 0.82 & 0.83 & 0.84 & 0.85 & 0.86 & 0.87 & 0.88 & 0.89 & 0.9 \\
0.71 & 1 & 0.73 & 0.74 & 0.75 & 0.76 & 0.77 & 0.78 & 0.79 & 0.8 & 0.81 & 0.82 & 0.83 & 0.84 & 0.85 & 0.86 & 0.87 & 0.88 & 0.89 & 0.9 \\
0.71 & 0.72 & 1 & 0.74 & 0.75 & 0.76 & 0.77 & 0.78 & 0.79 & 0.8 & 0.81 & 0.82 & 0.83 & 0.84 & 0.85 & 0.86 & 0.87 & 0.88 & 0.89 & 0.9 \\
0.71 & 0.72 & 0.73 & 1 & 0.75 & 0.76 & 0.77 & 0.78 & 0.79 & 0.8 & 0.81 & 0.82 & 0.83 & 0.84 & 0.85 & 0.86 & 0.87 & 0.88 & 0.89 & 0.9 \\
0.71 & 0.72 & 0.73 & 0.74 & 1 & 0.76 & 0.77 & 0.78 & 0.79 & 0.8 & 0.81 & 0.82 & 0.83 & 0.84 & 0.85 & 0.86 & 0.87 & 0.88 & 0.89 & 0.9 \\
0.71 & 0.72 & 0.73 & 0.74 & 0.75 & 1 & 0.77 & 0.78 & 0.79 & 0.8 & 0.81 & 0.82 & 0.83 & 0.84 & 0.85 & 0.86 & 0.87 & 0.88 & 0.89 & 0.9 \\
0.71 & 0.72 & 0.73 & 0.74 & 0.75 & 0.76 & 1 & 0.78 & 0.79 & 0.8 & 0.81 & 0.82 & 0.83 & 0.84 & 0.85 & 0.86 & 0.87 & 0.88 & 0.89 & 0.9 \\
0.71 & 0.72 & 0.73 & 0.74 & 0.75 & 0.76 & 0.77 & 1 & 0.79 & 0.8 & 0.81 & 0.82 & 0.83 & 0.84 & 0.85 & 0.86 & 0.87 & 0.88 & 0.89 & 0.9 \\
0.71 & 0.72 & 0.73 & 0.74 & 0.75 & 0.76 & 0.77 & 0.78 & 1 & 0.8 & 0.81 & 0.82 & 0.83 & 0.84 & 0.85 & 0.86 & 0.87 & 0.88 & 0.89 & 0.9 \\
0.71 & 0.72 & 0.73 & 0.74 & 0.75 & 0.76 & 0.77 & 0.78 & 0.79 & 1 & 0.81 & 0.82 & 0.83 & 0.84 & 0.85 & 0.86 & 0.87 & 0.88 & 0.89 & 0.9 \\
0.71 & 0.72 & 0.73 & 0.74 & 0.75 & 0.76 & 0.77 & 0.78 & 0.79 & 0.8 & 1 & 0.82 & 0.83 & 0.84 & 0.85 & 0.86 & 0.87 & 0.88 & 0.89 & 0.9 \\
0.71 & 0.72 & 0.73 & 0.74 & 0.75 & 0.76 & 0.77 & 0.78 & 0.79 & 0.8 & 0.81 & 1 & 0.83 & 0.84 & 0.85 & 0.86 & 0.87 & 0.88 & 0.89 & 0.9 \\
0.71 & 0.72 & 0.73 & 0.74 & 0.75 & 0.76 & 0.77 & 0.78 & 0.79 & 0.8 & 0.81 & 0.82 & 1 & 0.84 & 0.85 & 0.86 & 0.87 & 0.88 & 0.89 & 0.9 \\
0.71 & 0.72 & 0.73 & 0.74 & 0.75 & 0.76 & 0.77 & 0.78 & 0.79 & 0.8 & 0.81 & 0.82 & 0.83 & 1 & 0.85 & 0.86 & 0.87 & 0.88 & 0.89 & 0.9 \\
0.71 & 0.72 & 0.73 & 0.74 & 0.75 & 0.76 & 0.77 & 0.78 & 0.79 & 0.8 & 0.81 & 0.82 & 0.83 & 0.84 & 1 & 0.86 & 0.87 & 0.88 & 0.89 & 0.9 \\
0.71 & 0.72 & 0.73 & 0.74 & 0.75 & 0.76 & 0.77 & 0.78 & 0.79 & 0.8 & 0.81 & 0.82 & 0.83 & 0.84 & 0.85 & 1 & 0.87 & 0.88 & 0.89 & 0.9 \\
0.71 & 0.72 & 0.73 & 0.74 & 0.75 & 0.76 & 0.77 & 0.78 & 0.79 & 0.8 & 0.81 & 0.82 & 0.83 & 0.84 & 0.85 & 0.86 & 1 & 0.88 & 0.89 & 0.9 \\
0.71 & 0.72 & 0.73 & 0.74 & 0.75 & 0.76 & 0.77 & 0.78 & 0.79 & 0.8 & 0.81 & 0.82 & 0.83 & 0.84 & 0.85 & 0.86 & 0.87 & 1 & 0.89 & 0.9 \\
0.71 & 0.72 & 0.73 & 0.74 & 0.75 & 0.76 & 0.77 & 0.78 & 0.79 & 0.8 & 0.81 & 0.82 & 0.83 & 0.84 & 0.85 & 0.86 & 0.87 & 0.88 & 1 & 0.9 \\
0.71 & 0.72 & 0.73 & 0.74 & 0.75 & 0.76 & 0.77 & 0.78 & 0.79 & 0.8 & 0.81 & 0.82 & 0.83 & 0.84 & 0.85 & 0.86 & 0.87 & 0.88 & 0.89 & 1
\end{bmatrix}
$$

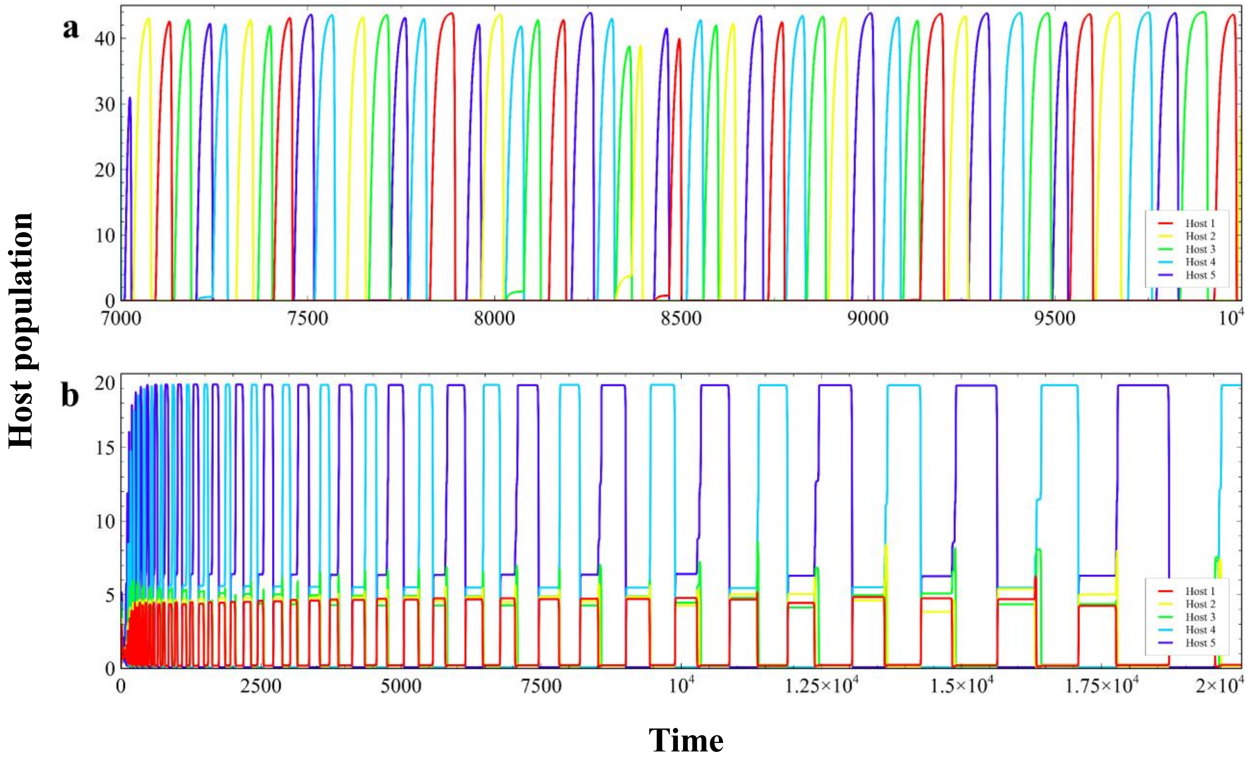

Supplementary Fig. 7. Red Queen dynamics occurring in modified deterministic host-parasite interaction model. The host population time series are shown in the figure. (**a**) Red Queen cycles emerge in host-parasite interaction model with non-polynomial inter-host competition. (**b**) In using type-III functional response, it is possible that exactly two host types generate the Red Queen cycles. The rest of the host types do not exhibit Red Queen cycles.

Notes:

In Supplementary Fig. 7a, the host-parasite interaction model with non-polynomial inter-host competition is as follows:

$$\frac{dH_i}{dt} = \left(\left(\frac{r_i}{1+\sum_{k=1}^{5} H_k} K_i - \delta_i \right) - \sum_{k=1}^{5} \frac{\alpha_{ik}}{1+H_i} P_k \right) H_i \qquad \text{(Eq. S8)}$$

$$\frac{dP_j}{dt} = \left(\sum_{k=1}^{5} \frac{\alpha_{kj} H_k}{1+H_k} - d_j \right) P_j \qquad \text{(Eq. S9)}$$

$i, j = 1,2,\ldots,5$.

Here, the host growth rate coefficient r_i is not necessarily the same as the r_i in Eq. S5. As inter-host competition increases, the effect of the host growth rate coefficient is reduced. The competition among the hosts is represented by a rational function, where r_iK_i/δ_i is an upper

bound of the equilibrium value of H_i. The parameter K_i affects the upper bound, hence influences the carrying capacity of each host type. The parameter δ_i denotes host death rate that is not due to parasitism. For further mathematical details about the non-polynomial inter-host competition model, see Rabajante and Talaue (2015) in the list of references.

The parameter values are $r_i=0.9+0.01(i-1)$, $K_i=5$ for all i, $s_i=0.1+0.001(i-1)$, $d_j=0.15+0.01(i-1)$, $\alpha_{ij}=0.01$ for $i\neq j$ and $\alpha_{ij}=0.96$ for $i=j$. Without parasitism, the host population converges to an equilibrium point.

In Supplementary Fig. 7b, the model is as follows:

$$\frac{dH_i}{dt}=\left(r_i\left(1-\frac{\sum_{k=1}^{5}H_k}{K}\right)-\sum_{k=1}^{5}\frac{\alpha_{ik}H_i}{1+H_i^2}P_k\right)H_i \qquad \text{(Eq. S10)}$$

$$\frac{dP_j}{dt}=\left(\sum_{k=1}^{5}\frac{\alpha_{kj}H_k^2}{1+H_k^2}-d_j\right)P_j \qquad \text{(Eq. S11)}$$

$i, j = 1,2,\ldots,5$.

The parameter values are $r_i=0.95+0.01(i-1)$, $K=20$, $d_j=0.475+0.01(i-1)$, $\alpha_{ij}=1/15$ for $i\neq j$ and $\alpha_{ij}=11/15$ for $i=j$.
